# A Study on Factors That Drive Variation in the Levels of Social Capital Among People Living With HIV/AIDS in Iran

**DOI:** 10.5539/gjhs.v7n3p351

**Published:** 2015-03-30

**Authors:** Shahnaz Rimaz, Zahra Nikooseresht, Samera Vesali, Saharnaz Nedjat, Mohsen Asadi-Lari

**Affiliations:** 1 Department of Epidemiology, School of Public Health, Iran University of Medical Sciences, Tehran, Iran; 2 School of Public Health, Tehran University of Medical Sciences, Tehran, Iran; 3 Department of Epidemiology & Biostatistics, School of Public Health, Knowledge Utilization Research Center, Tehran University of Medical Sciences, Tehran, Iran; 4 Oncopathology Research Centre, Iran University of Medical Sciences, Tehran, Iran

**Keywords:** social capital, HIV/AIDS, World Bank, Social Capital Questionnaire (SC-IQ)

## Abstract

**Introduction:**

Social capital is increasingly used in relation to health issues, particularly in sexually transmitted diseases/infections and health behaviors. Experiences indicated that social capital can contribute in changing HIV related risk behaviors and a decline of HIV infection through social groups and networking and make more effective use of HIV/AIDS prevention, care, and treatment services. We aimed to assess social capital in these persons through a quantitative study.

**Method:**

This cross-sectional study was performed with a convenience sample of 300 people living HIV/AIDS referred to a counseling center of behavioral diseases, in Imam Khomeini Hospital, in Tehran, the capital of Iran, during September 2011 to May 2012.

Data collection tools were a demographic questionnaire and World Bank Social Capital Questionnaire (SC-IQ). The analysis of data was performed by the SPSS statistic software version 18. To identify factors influencing social capital in participations, Pearson correlation coefficient, ANOVA, t-test, and a multiple regression were applied. The significant level was considered 0.05 in this study.

**Results:**

165 (55%) were male and the rest female. The mean age of participants was 34.3±7.5. The mean score of total social capital was 2.34±0.5 in all participants. The domain of individual trust had the highest mean score (2.53±0.66). The lowest mean score was related to the domain of social trust and associative relations (2.23±0.62). Variables such as ethnicity, age, and middle economic status had a significant impact on the domain of individual trust so that the mean score of this component of social capital was lower among women (0.396) than men. Factors affecting total social capital were ethnicity and middle economic status.

**Conclusion:**

Finding emphasized on the role of economic status, ethnicity and gender in persons living with HIV/AIDS. Thus recommended that policy makers and program managers consider social groups and networks, especially in women in the design and delivery of intervention strategies to reduce HIV transmission.

## 1. Introduction

Many studies demonstrated that determinants of health and well being are complex, and reflect a mix of economic, environmental, social, biological, and genetic factors. Also, it is emphasized on social determinants of public health (Baumet al., 1999; Gregson et al., 2011; Lynch, Due, Muntaner, & Smith, 2000), and that social capital might play an important role in improving health (Gregson et al., 2011; Baum, 2000; Campbell, Williams, & Gilgen, 2002; Lomas, 1998; Moore, Haines, Hawe, & Shiell, 2006). According to Putnam’s definition, social capital includes components like the levels of social and civic trust, the presence of both thick and thin or embedded and autonomous networks, and coordination and cooperation for mutual benefit (Baum, 1999; Pronyk, Harpham, T. Busza, Phetla, Morison, Hargreaves, Kim, Watts & Porter, 2008).

Researchers showed that components of social capital, especially the social networks and contribution of individuals in communities, have high impacts on health care and outcomes. As well, it is a potentially useful construct for understanding health disparities (Campbell, Williams, & Gilgen, 2002; Cené et al., 2011).

Social capital is increasingly used in relation to health issues, particularly in sexually transmitted diseases/infections and health behaviors (Pronyk, Harpham, Busza, Phetla, Morison, Hargreaves, Kim, Watts & Porter, 2008; Cené et al., 2011; Siahpush, & Singh, 2000). There has been stigmatizing attitudes to HIV/AIDS in many societies (Chiu et al., 2008). Experiences indicated that social capital can contribute in changing HIV related risk behaviors and a decline of HIV infection through social groups and networking (Frumence et al., 2010), and make more effective use of HIV/AIDS prevention, care, and treatment services (Gregson et al., 2011).

Based on the UNAIDS (United Nations Program on HIV/AIDS) report, 35.3 million [32.2 million–38.8 million] people were living with HIV at the end of 2012, globally. Worldwide, the number of people newly infected was (2.3 million [1.9 million–2.7 million]) in 2012. Since 2001, the number of people newly infected in the Middle East and North Africa has increased by more than 35% (from 27 000 [22 000–34 000] to 37 000 [29 000–46 000]) (UNAIDS, 2012). In Iran, there were 26125 PLWIH, which 5182 of them died of the disease, in 2011 (Ministry of Health of Iran, HIV/AIDS status in Iran, 2012).

Considering that most existing studies on social capital in PLWIH are either qualitative, also because the founders and legislators require quantitative evidence of a program’s success to allocate financial resources (Hawley et al., 2012), we aimed to assess social capital in these persons through a quantitative study.

## 2. Method

This cross-sectional study was carried out on 300 people living HIV/AIDS referred to a counseling center of behavioral diseases, in Imam Khomeini Hospital, in Tehran, the capital of Iran, during September 2011 to May 2012.

Sampling was done as convenient, and the sample size was calculated by the formula related to cross-sectional studies:


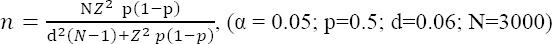


Finally, 300 subjects participated in the study. Also, patients were included into the study if they met these criteria: 1) HIV positive, 2) registered at the consulting clinic, 3) aged more than 18 years. Exclusion criteria were lack of cognitive, communicative disabilities, or psychotic disorders. There was no limitation for the stage or the length of the disease; i.e. Patients were recruited regardless of the stage and duration of HIV/AIDS infection.

Ethical approval for the study was obtained from Tehran University of Medical Sciences. A written consent was duly obtained from the participants to ensure that they participated voluntarily. Both questions and answers were read out to the patients and the questionnaire was completed by a trained administrator.

### 2.1 Data Collection

Data collection tools were as follows:

1) a demographic questionnaire included variables such as: gender, age, ethnicity, marital status (single, married), the level of education (primary and middle School, high school and diploma, higher than diploma), occupational status (employed, unemployed), economic status (poor, middle, rich) according to monthly mean income, and information related to the disease (duration, transmission mode, clinical stage.

2) World Bank Social Capital Questionnaire (SC-IQ) (Adjusted form related to literature for assessing social capital in Iran (Ashrafi et al., 2012; Asadi-Lari1 et al., 2010): This questionnaire was included 9 main questions and 3 components as follows: 1) individual trust, 2) cooperation and social support, 3) social trust and associative relations. Each domain had questions in the form of Likert, multiple choice or Yes/No.

To determine the participants’ social capital components in this study, we calculated each person’s score in each domain. The attainable score was 1–5 in each domain. Participants were ranked based on attainable score as below:

**Table:** 

Ranking	Score
Very high	<4
High	3.01 - 4
Medium	2.01- 3
Low	1 - 2

### 2.2 Data Analysis

The analysis of data was performed by the SPSS statistic software version 18. To analyze, descriptive statistics such as frequency tables, mean, and standard deviation was used.

To identify factors influencing social capital in participations, Pearson correlation coefficient, ANOVA, t-test, and a multiple regression were applied. The significant level was considered 0.05 in this study.

## 3. Results

### 3.1 Demographic and Clinical Factors of Participants

Out of 300 participants, 165 (55%) were male and the rest female. The mean age of participants was 34.3±7.5. More than half of the subjects were 35 years old or younger (56%), and 57% were married. 48.7% of them had a high school education, and 53.7% were employed. The majority were aware of their disease less than 5 years (64.7%). Most of them were (51.7%) HIV positive and a symptomatic and others were associated with AIDS. The most common transmission mode was an intravenous drug injection addiction in men (52.7%) and unprotected intercourse in women (60.0%) (Table 1).

**Table 1:** Demographic characteristic of study participants (n=300)

Variable		Male		Female		Total	

		Frequency	Percent	Frequency	Percent	Frequency	Percent
**Age groups**	≤35	83	50.3	85	63.0	168	56
	>35	82	49.7	50	37.0	132	44
**Education level**	Primary and Middle School	56	33.9	43	31.9	99	33.0
	High school and diploma	81	49.1	65	48.1	146	48.7
	Higher than diploma	28	17.0	27	20.0	55	18.3
**Marital status**	Married	80	48.5	91	67.4	171	57.0
	Single	85	51.5	44	32.6	129	43.0

**Occupational status**	Employed	130	78.8	31	23.0	161	53.7
	Unemployed	35	21.2	104	77.0	139	46.3
**Duration of disease**	<5 years	109	66.1	85	83.0	194	64.7
	≥5 years	56	33.9	50	37.0	106	35.3
**Clinical stage**	HIV	52	49.7	73	54.1	155	51.7
	AIDS	83	50.3	62	45.9	145	48.3
**Transmission mode**	Blood and Intravenous drug addiction	87	52.7	14	10.4	101	33.7
	Sexual contact	37	22.4	81	60.0	118	39.3
	Mother to child	2	1.2	1	0.7	3	1.0
	Unknown	39	23.6	39	28.9	78	26.0

### 3.2 Social Capital Domains

The mean score of total social capital was 2.34±0.5 in all participants. The domain of social trust and associative relations had the lowest mean score (2.23±0.62). The highest mean score was related to the domain of Individual trust (2.53±0.66) (Table 2).

**Table 2:** Scores related to social capital and it’s domains in the study population (n=300)

Social capital	Minimum-maximum scores	Mean (Standard deviation)
Individual trust	1.44-4.33	2.53(0.66)
Cooperation and social support	1.11-3.53	2.26(0.53)
Social trust and associative relations	1.00-4.20	2.23(0.62)
Total social capital	1.28-3.75	2.34(0.50)

### 3.3 Factors Affecting Social Capital and Its Each Domain

To assess the correlation between independent variables and social capital (mean scores of domains and the overall mean of social capital), Pearson correlation coefficient, ANOVA and t-test were used. The variables entered into regression models had a p-value <0.2. As can be seen in Table 3, variables such as ethnicity, gender, and middle economic status had a significant impact on the domain of individual trust so that the mean score of this component of social capital was lower among women (0.396) than men, and higher in participants with ethnicity “Fars” and middle economic status. Factors like ethnicity and rich and middle economic status were effective on cooperation and social support. In other words, PLWIH with ethnicity “Fars” and middle and economic status had mean greater social capital into this domain. Factors affecting total social capital were ethnicity and middle economic status.

**Table 3:** Association between demographic characteristics and social capital based on a multiple regression

Variable		β	SE	t	P-value
Individual trust	Ethnicity	0.215	0.093	2.319	0.022
	gender	-0.396	0.114	-3.483	0.001
	Middle economic status	0.191	0.094	2.036	0.043
Cooperation and social support	Ethnicity	0.200	0.074	2.717	0.007
	Middle economic status	0.316	0.087	3.648	≤0.0001
	Rich economic status	0.294	0.122	2.407	0.017
Social trust and associative relations		-	-	-	-
Total social capital	Ethnicity	0.178	0.075	2.357	0.020
	Middle economic status	0.175	0.076	2.290	0.023

## 4. Discussion

In recent years, researches have been developed to evaluate the role of social capital in public health and health promotion (Smith & Lynch, 2004; Muntaner, 2004; Szreter & Woolcock, 2004).

This was the first study to measure the components of social capital in PLWIH in Iran. It was performed in individual-level, which provide the most simple and clearly defined units of measurement, and avoids the problems associated with the use of aggregated data. Also, decision making for investing in social capital take place generally by individuals not communities (Ma, Qin, Chen, Li, & Ren Chen, 2012).

HIV/AIDS has caused a serious socioeconomic impact like poverty, declining income- social exclusion and stigma and a threat to national security (Pronyk, Harpham, Morison, Hargreaves, Kim, Phetla, Watts, & Porter, 2008; Muriisa & Jamil, 2011). “Therefore, the relationship between social capital and health may be different among PLWIH in comparison with more general population. Globally, PLWH are highly marginalized” (Webel et al., 2012, p. 2).

A large number of studies (Campbell et al., 2013; Webel et al., 2012; Cené et al., 2011) implemented on social capital and HIV, particularly in South Africa (in rural regions) (Cené et al., 2011). They had an emphasis on preventing transmission of HIV and showed that social networks can help with discounting high-risk behaviors, especially in intravenous drug injection users or sex workers in some of developing societies. Through disseminating health related information and show-case positive role modeling behaviors such as condom use or access to HIV testing, and shape community norms around gender relations, communication and sexual negotiation.

In the other words, strong social networks may apply social or cultural pressure to cause ban high-risk sexual activities even for people with HIV. Individuals with positive HIV entered to “wider networks and deeper trust relationships may have a stronger sense of self-confidence, self esteem and may be better able to take control over decision making” (Pronyk, Harpham, Morison, Hargreaves, Kim, Phetla, Watts, & Porter, 2008 2008, p.2).

Also, the emotional support generated in these networks may reduce social isolation, discrimination and stigmatizing attitudes, namely inferior or inappropriate reactions due to fear or perceived threats, associated with negative effects on communicating about prevention, negotiating condom use and seeking an HIV test and create a more accepting environment for those living with the disease (Pronyk, Harpham, Morison, Hargreaves, Kim, Phetla, Watts, & Porter, 2008; Muriisa & Jamil, 2011; Sivaram et al., 2009). Other studies declared that group membership had a particularly protective effect against HIV infection for women (Campbell et al., 2013). Thus, these components of social capital can have preventive effects on incidence and prevalence of HIV/AIDS. In contrast, lack of social capital and absence of community cohesion and the transformation of social associated with chronic poverty played a major role in limiting the impact of an effectiveness and well-conceived intervention program (Pronyk, Harpham, Morison, Hargreaves, Kim, Phetla, Watts, & Porter, 2008).

As can be showed in this study, one of variables influencing domains of social capital is economic status so that the economic status better, the mean score of social capital domains higher in PLWIH. Also, globally, Poverty as a major factor for many of the world’s most marginalized PLWIH challenged and researches outcome shows that wealth inequality is a significant factor to explain the risk of HIV in the community (Webel et al., 2012).

Our study indicated total social capital and its components achieved medium rank (mean score >2) in people living with HIV/AIDS referred to this counseling center of behavioral diseases. Communities with high levels of social capital, lower mortality rates and higher life expectancy and less violence (Pattussi, Moysés, Junges, & Sheiham, 2006). As well as, social capital can clarify the role and implications of the social environment on health outcomes among PLWIH, and its ‘‘features of social organization, such as trust, norms and networks that can improve the efficiency of society by facilitating coordinated action’’ (Putnam et al., 1993).

## 5. Limitations

Our study has some limitations. First, since the design of this study was cross-sectional, an association founded between social capital and other variables did not show causal relationships. Therefore, it is required to conduct interventional studies to enhance efficacy of each domain in health promotion and decline adverse effects resulting from behavioral diseases. Also, to understand the details of the problems related to HIV/AIDS patients, qualitative researches should be designed and done.

Second, the results may not be generalized to all Iranian HIV/AIDS patients. Our data were collected in the counseling center of behavioral diseases, in Imam Khomeini Hospital, in Tehran, the capital of Iran.

## 6. Conclusion

Findings emphasized on the role of social capital and its domains in public health and infectious diseases in particular HIV/AIDS. Social capital is able to decrease transmission and the burden of disease, high risk behaviors of PLWIH, and control them through trust and social relations.
